# Optoelectronic Performance Variations in InGaN/GaN Multiple-Quantum-Well Light-Emitting Diodes: Effects of Potential Fluctuation

**DOI:** 10.3390/ma11050743

**Published:** 2018-05-07

**Authors:** Abu Bashar Mohammad Hamidul Islam, Jong-In Shim, Dong-Soo Shin

**Affiliations:** 1 Department of Electronics and Communication Engineering, Hanyang University ERICA, Ansan 15588, Korea; hamidul@spl.hanyang.ac.kr (A.B.M.H.I.); jishim@hanyang.ac.kr (J.-I.S.); 2 Department of Photonics and Nanoelectronics and Department of Bionanotechnology, Hanyang University ERICA, Ansan 15588, Korea

**Keywords:** light-emitting diodes, strain, piezoelectric field, point defects, potential fluctuation, carrier localization

## Abstract

We investigate the cause of the optoelectronic performance variations in InGaN/GaN multiple-quantum-well blue light-emitting diodes, using three different samples from an identical wafer grown on a *c*-plane sapphire substrate. Various macroscopic measurements have been conducted, revealing that with increasing strain in the quantum wells (QWs), the crystal quality improves with an increasing peak internal quantum efficiency while the droop becomes more severe. We propose to explain these variations using a model where the in-plane local potential fluctuation in QWs is considered. Our work is contrasted with prior works in that macroscopic measurements are utilized to find clues on the microscopic changes and their impacts on the device performances, which has been rarely attempted.

## 1. Introduction

Nitride-based material systems have been studied extensively to improve the optical properties for applications in various optoelectronic devices [1,2]. Especially, the InGaN-based multiple quantum wells (MQWs) can cover a wide spectral range from the ultraviolet to visible (blue and green) spectra [3]. Although the InGaN epitaxial layers grown on sapphire substrates typically show high dislocation densities (in the order of 10^9^ cm^−2^) resulting from the large lattice mismatch between GaN and the sapphire substrate [2], InGaN-based MQW light-emitting diodes (LEDs) show surprisingly high internal-quantum-efficiency (IQE) characteristics. It has been widely accepted through experiments and simulations that the injected charge carriers are spatially separated from defects owing to higher electrical potentials at the edges of the defects [3,4]. Consequently, carriers tend to be prevented from diffusing into the defects [3,4] and recombine radiatively in the mostly nondefective area. While the reasons for this carrier localization effect have been investigated by various research groups [4,5,6,7,8,9,10,11,12,13,14,15,16,17,18,19,20,21,22,23,24,25], the origin of the electrical potential fluctuation still remains a controversy. Such factors as alloy (especially In) fluctuation [5,6,7,8,9,10,11,12], thickness variation of the quantum wells (QWs) [10,11,12,13,14], In-N-In chains [15,16], In clustering [17,18], crystallographic defects [19,20,21,22,23], crystal quality [24], the piezoelectric field due to strain [10,21,25], and any combined effects have been pointed out as possible reasons. Due to the quantum size effect, their influence on the electrical potential fluctuation would be more enhanced in the QWs than in the bulk layer.

In general, carriers have a natural tendency to stay in the lower energy region, which indicates that the carriers are preferably localized in the lower potential energy of the fluctuating energy bandgap. This kind of carrier localization reduces the effect of nonradiative recombination at the defects [4,15] and increases the radiative recombination. Defects (especially point defects) act as nonradiative recombination centers (NRCs) or tunneling paths for the carriers, which cause the reduction of the radiative recombination rate at low currents [26,27,28]. The amount of the radiative recombination rate in LEDs is determined by the IQE, which is the ratio of the radiative recombination rate to the total recombination rate [26]. Thus, the radiative and nonradiative current components of the sample can be separated by using the IQE data of the sample [28].

In addition to the defects, InGaN/GaN QWs grown on a *c*-plane sapphire substrate experience an internal electric field on top of the built-in field in the depleted *pn* junction. The internal field is caused by the difference in polarization, especially the piezoelectric polarization [29,30]. The overall electric field in the QWs causes the quantum-confined Stark effect (QCSE) [31]. The strength of this piezoelectric field in blue LEDs is quite high, above ~1 MV/cm [29,30]. Consequently, the electron-hole wavefunctions are separated from each other, resulting in a reduction of the radiative recombination rate in LEDs. It also reduces the effective active volume of the QWs and can eventually cause the carrier overflow from the active QWs. The blue shift of the electroluminescence (EL) peak with increasing current is caused by the combined effect of the piezoelectric-field screening and the band-filling of the localized states [32,33,34,35].

Various nonradiative recombination processes are considered available in or around the active QW region of the LED, including (i) the Shockley-Read-Hall (SRH) recombination in the active QWs; (ii) overflown electrons recombining in the p-type clad layer; (iii) electron tunneling via defects from the active QWs to the p-type layer; and (iv) the Auger recombination [36,37]. The so-called efficiency droop occurs only when the nonradiative recombination processes become faster than the radiative recombination process with increasing current [36].

In order to elucidate the reason for the high efficiency of the InGaN-based QWs despite the high-density defects, a significant amount of studies have been conducted on the recombination of localized carriers at certain potential-energy minima originating from the bandgap variation [8,9,10,11,12,13,14,15,16,17,18,19,20,21,22,23,24,25,32]. The local bandgap variation in the QWs has often been correlated with defect-originated microstructures [19,20,21,22,23] that prevent the carriers from diffusing into defects and cause the radiative recombination in mostly nondefective areas [4,25]. In this respect, the local variation of the bandgap energy in the QWs due to inhomogeneous defect distributions [19,20,21,22,23] (especially point defects in the QWs) and strain [10,21,25,38] need to be studied in order to understand the high efficiency of InGaN-based LEDs. However, most of the previous research works have been focused on the material (or microscopic) properties rather than the device performances. Therefore, there exists a definite need to examine the influences of the local potential-energy fluctuation on the macroscopic device performances in more detail.

In this paper, we utilize InGaN/GaN MQW blue LEDs that have been selected from an identical wafer grown on a *c*-plane sapphire substrate. By comparing their macroscopic electrical and optical performances, we demonstrate the relationships among various factors, such as strain, defect density, IQE, and the IQE droop. We show, systematically, how the local in-plane potential-energy fluctuation, influenced by the inhomogeneous defect distribution and the piezoelectric field in InGaN/GaN MQWs, interactively affects the device performances.

## 2. Samples and Experiments

Three different LED chips were selected from an identical wafer, labeled as samples #1, #2, and #3. All samples had undergone the identical epitaxial growth and fabrication processes. Any small differences in electrical and optical properties among the samples resulted from the nonuniformity in epitaxial growth conditions and/or fabrication processes across the wafer. The basic epitaxial structure of the LED samples consisted of a Si-doped n-GaN layer (doping concentration = 1 × 10^19^ cm^−3^), a five-period MQW active region (2.4 nm thick InGaN wells and 4.8 nm thick GaN barriers, except the 9.6 nm thick last barrier), and a 120 nm thick p-type GaN clad layer, successively grown on a *c*-plane sapphire substrate by using metal-organic chemical vapor deposition. The In composition of InGaN well layers of all samples was estimated to be ~13%. The chip size was 600 × 700 μm^2^ with the lateral-electrode structure. The chips were mounted on TO-type metal packages without epoxy molding for measuring both electrical and optical characteristics.

Light output power vs. current (*L-I*) was measured by an integrating sphere system (CAS 140CT, Instrument Systems, Munich, Germany) and the EL intensity was measured by a Si p-i-n photodiode. The current-voltage (*I-V*) characteristics were measured by a Keithley semiconductor parameter analyzer (Keithley, Cleveland, OH, USA) with a sweep rate of 0.35 Vs^−1^. A fiber-optic spectrometer (AvaSpec-2048, Avantes, Apeldoorn, The Netherlands) was used to find the emission spectrum and peak-wavelength characteristics of all LED samples. The capacitance-voltage (*C-V*) characteristics were measured by an Agilent 4284A LCR meter (Agilent, Santa Clara, CA, USA) at a frequency of 1 MHz with an AC modulating voltage of 10 mV for calculating the depletion width of all samples. In the electroreflectance (ER) spectroscopy, the output from a 150 W xenon arc lamp was imposed on a dual-grating monochromator (DK240, Spectral Products, Putnam, CT, USA) to generate the probe beam that vertically illuminated the sample. For the phase-sensitive detection, a lock-in amplifier (SR810, Stanford Research Systems, Sunnyvale, CA, USA) was used. A function generator with a voltage of 100 mV at 500 Hz was applied to the samples for modulating the internal electric field and changes in reflectivity *R*, i.e., Δ*R*, was measured as a function of the wavelength.

Table 1 summarizes various optoelectronic characteristics of the samples. The differences among the samples are analyzed in detail in the next section. While the changes in various optoelectronic performances are minute, the overall trend reflects the actual changes in MQWs of the samples.

## 3. Results and Discussion

### 3.1. Experimental Results from the LED Samples

**Figure 1:**
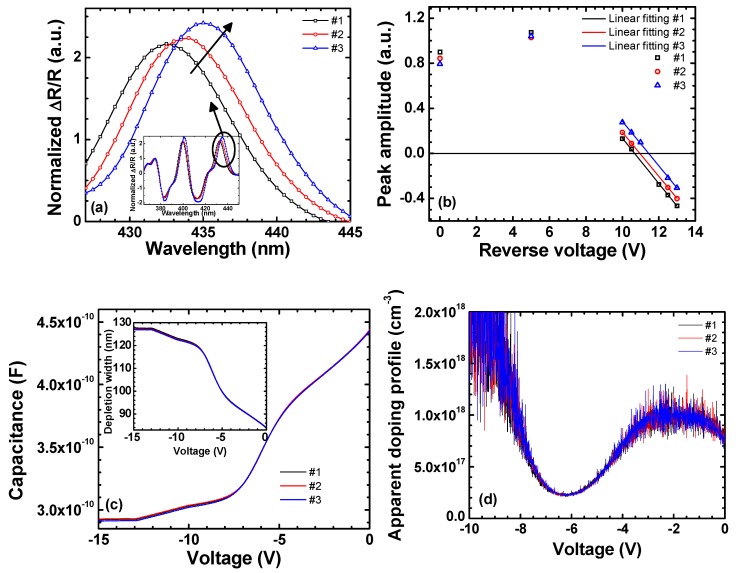
(**a**) Normalized ER spectra measured at a bias of 0 V. The inset shows the ER spectra from 360 to 460 nm. (**b**) The peak amplitude at the MQW region as a function of applied reverse bias. (**c**) The *C-V* characteristics. The inset shows the calculated depletion width. (**d**) The apparent doping profile obtained from (**c**).

Figure 1a shows the ER spectra at a bias of 0 V for the three samples. As shown in the inset, the spectra have been normalized at their peak intensities around 370 nm, for which the GaN barrier layers are responsible. The normalization around 370 nm assumes that the GaN layers are identical for these samples, which is confirmed by the fact that the peak position rarely moves: the dominant cause of LED performance variations in our study resides in the InGaN wells. It is seen in Figure 1a that the peak amplitude of the ER spectrum near the QW emission (~435 nm) increases with its peak wavelength. The differences in peak amplitude of the ER spectra indicate that the QWs undergo slightly different strains. We compare the piezoelectric field for these three samples by the following three-step experiments: (1) We measure the ER spectrum under various reverse bias conditions; (2) we find the flat-band voltage, at which the sign of the peak amplitude around 435 nm is reversed, and the depletion width at this flat-band voltage; and (3) we calculate the piezoelectric field [39]. The measured flat-band voltages for samples #1, #2, and #3 are −10.5, −10.9, and −11.5 V, respectively, which are obtained by using the *x*-intercepts of the linearly-fitted peak amplitudes of the bias-dependent ER spectra, as shown in Figure 1b [40]. The depletion widths are obtained by using the *C-V* characteristics as shown in Figure 1c [41]. The measured capacitances and the calculated depletion widths of these samples are almost the same owing to the fact that the samples are from the identical wafer. The depletion widths obtained in this way at their *V*_FB_ are 120.0, 124.1, and 124.6 nm for #1, #2, and #3, respectively. Figure 1d shows the apparent doping profiles obtained from the data shown in Figure 1c. It is observed that the profiles are almost identical for the samples under study.

The final piezoelectric fields obtained for samples #1, #2, and #3 are –1.35, –1.40, and –1.50 MV/cm, respectively, as summarized in Table 1. The variation of the piezoelectric field across the wafer is considered due to the inhomogeneous defect distribution, which causes the lattice to relax differently. This point is discussed in detail later. There is a possibility of inhomogeneous In composition across the wafer, but it is difficult to discriminate the two effects at the moment. There may still be the correlation between the defect distribution and the In incorporation: the combined effects might be behind the observation.

**Figure 2:**
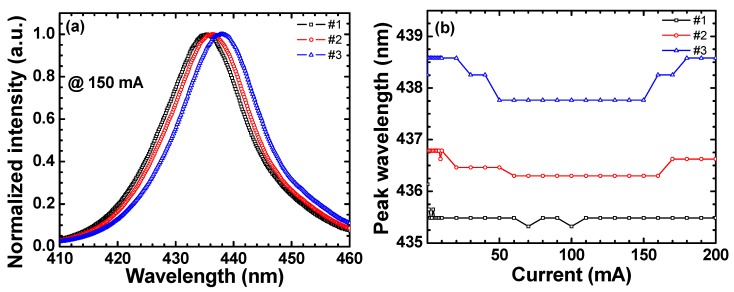
(**a**) Normalized EL spectra at 150 mA and (**b**) peak wavelengths as a function of current.

Figure 2a shows the normalized EL spectra at 150 mA, where the peak wavelengths are 435.5, 436.5, and 438.0 nm for samples #1, #2, and #3, respectively, which is consistent with Figure 1a. The variation in the peak wavelength could be caused by the variation in such factors as the growth temperature [42]. From both the ER and EL spectra, it is considered that the strain slightly increases with the sample number. Figure 2b shows the peak wavelength as a function of current for all samples. Usually, the blue shift at small currents mainly results from the compensation of the QCSE and the band-filling of the localized states [32,33,34,35,36,37]. Sample #3 shows the highest blue shift with increasing current from zero, which is understood as the largest screening of the piezoelectric field among the three samples [32,34]. The red shift of these samples is described in the next section.

**Figure 3:**
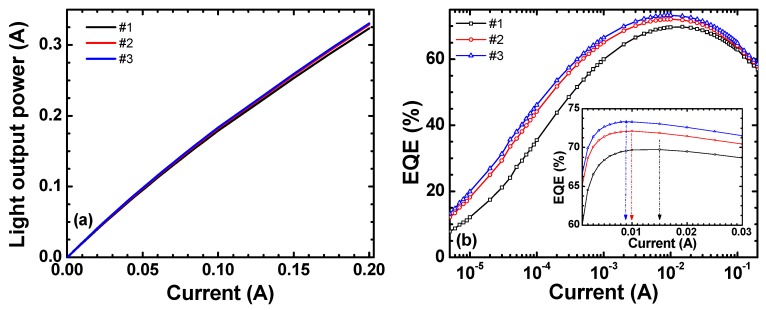

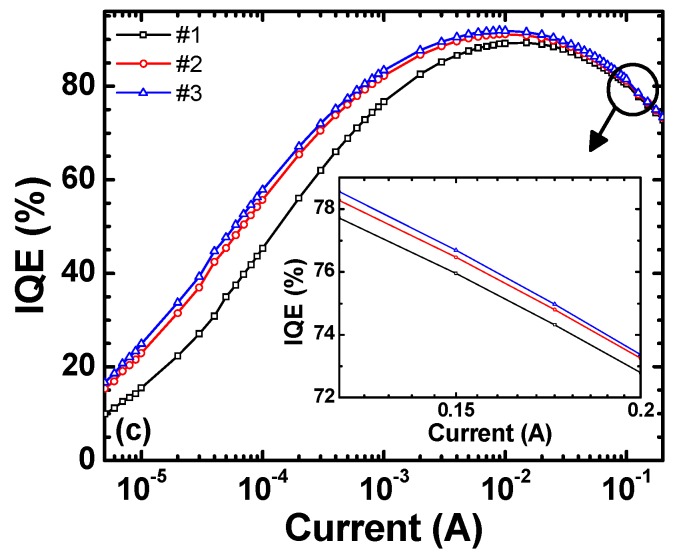
(**a**) *L-I* characteristics measured at room temperature, (**b**) EQE characteristics as function of current with the inset showing the EQE from 1 to 30 mA, and (**c**) IQE characteristics on semi-log scales. The inset shows the IQEs for the current from 0.125 to 0.200 A.

Differences in carrier recombination properties can be understood directly by analyzing the IQE characteristics. Figure 3a shows the *L-I* characteristics of the LED samples measured at room temperature by using an integrating sphere. The external quantum efficiency (EQE) is defined as the ratio of the number of emitted photons into free space per second to the number of electrons injected into the LED per second. Figure 3b shows the experimentally-calculated EQE as a function of current from the *L-I* characteristics [26]. The inset shows the EQE for a current of 1 to 30 mA to identify the current at the EQE peak. The IQE of these samples shown in Figure 3c are obtained by a method based on the improved carrier rate equation [43]. Both the EQE and IQE peaks are seen to increase with the sample number. For the case of samples with the identical epitaxial structure, the increase in the radiative recombination rate at low currents before the IQE peak is due to the decrease in defects (especially the point defects in the active MQW region) since the defects act as NRCs for carriers, inducing the SRH recombination. In Figure 3c, IQEs of all samples increase at currents up to ~1.5 × 10^−2^ A (~2.82 V) due to the rapid increase in radiative recombination rate over nonradiative recombination rate. Note that this indicates that the crystal quality is improved with the sample number. As seen in the inset of Figure 3b, the current at the EQE peak (consequently at the IQE peak), *I*_max IQE_, for samples #1, #2, and #3 are 15.0, 10.0, and 9.0 mA, respectively. The IQE peaks at these currents are 89.0%, 91.5%, and 92.0%, respectively.

The amount of the IQE droop for each sample is calculated by using Equation (1):

(1) $$\eta_{\mathrm{droop}}\left(I_{\mathrm{drive}}\right)=\frac{\eta_{\mathrm{IQE}}\left(I_{\max\;\mathrm{IQE}}\right)\;-\;\eta_{\mathrm{IQE}}\left(I_{\mathrm{drive}}\right)}{\eta_{\mathrm{IQE}}\left(I_{\max\mathrm{IQE}}\right)}$$

The IQE droops at a driving current (*I*_drive_) of 0.15 A are 15.0%, 16.0%, and 16.6% for samples #1, #2, and #3, respectively. It is noted that the IQE droops of these samples increase with the sample number: the sample with the highest strain (#3) has the highest IQE droop, as shown in the inset of Figure 3c. Hence, the IQE droop is related with the piezoelectric field (*F*_PZ_), which decreases the effective active volume [44]. As a result, at high currents, more electrons overflow from the QWs and recombine nonradiatively at the p-GaN layer with increasing sample number.

**Figure 4:**
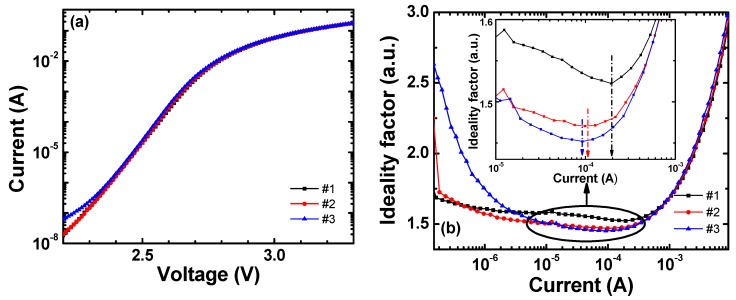
(**a**) *I-V* characteristics on semi-log scales and (**b**) calculated IFs from the *I-V* characteristics. The inset shows the ideality factor of all samples from 2.50 to 2.65 V.

The ideality factor (IF) from the *I-V* characteristics also has information on the recombination processes in an LED. We calculate the IF, *n*_ideal_, by using:

(2) $$n_{\mathrm{ideal}}=\frac{q}{k_{B}T}{\left(\frac{d\ln\;I}{dV}\right)}^{-1}$$

where *q* is the elementary charge, *k_B_* is the Boltzmann constant, and *T* is the absolute temperature [45]. Figure 4a shows the forward *I-V* curves near the current range where we calculate the IF. There is a slight increase in forward current at a given bias with the sample number. This is due to the increase in the total recombination rate, especially the radiative recombination rate, as the sample number increases.

Figure 4b shows the calculated IFs for all samples, where the minimum IF and the current at the minimum IF decrease with the sample number. The minimum IFs of samples #1, #2, and #3 are 1.52, 1.47, and 1.45, respectively, as shown in the inset of Figure 4b, and the current at the minimum IFs are 0.20, 0.10, and 0.09 mA for samples #1, #2, and #3, respectively. The band-to-band radiative recombination current has an IF of 1 [46], the nonradiative current associated with the SRH recombination via defects has an IF of 2, and the nonradiative recombination current from the defect-assisted tunneling or the surface recombination has an IF > 2 [47]. The decrease in the IF close to 1 indicates the rapid dominance of the radiative recombination rate over the nonradiative recombination rate. As the current increases from 5 × 10^−6^ to 5 × 10^−4^ A, the IF values become a minimum for all samples. The decrease in the minimum IF value represents that the number of defects (especially point defects) in the QWs decreases with the sample number. This analysis of the improved crystal quality with the sample number is consistent with the IQE characteristics. As the current increases >3 × 10^−4^, the IF steeply increases owing to the combined effects of series resistance in epitaxial layers and ohmic contacts. This part corresponds to the part where the bending occurs in the *I-V* characteristics on the semi-log scale.

### 3.2. Effects of Local Potential Fluctuation

We have seen that there exists a correlation between the strain, as confirmed by the piezoelectric field, and the defect density (or crystal quality), as indicated by the peak IQE and the minimum ideality factor. As the nitride-based material is grown on the *c*-plane sapphire substrate, there exist many threading dislocations [48], as well as large piezoelectric fields, in the growth direction [33]. In addition to the dislocations, it is considered that numerous point defects also exist in the active region caused by (i) vacancies or missing atoms such as Ga or N; (ii) interstitials, i.e., extra atoms not in regular lattice sites, such as Mg [49]; and (iii) anti-sites or the replacement of one kind of ion by another one [50]. These defects act as NRCs that trap carriers and also cause leakage of carriers by working as tunneling paths. Moreover, the point defects in the active region play an important role in creating the in-plane potential fluctuation in the QWs [19] since the point defects can change the uniform distribution of In atoms [51].

**Figure 5:**
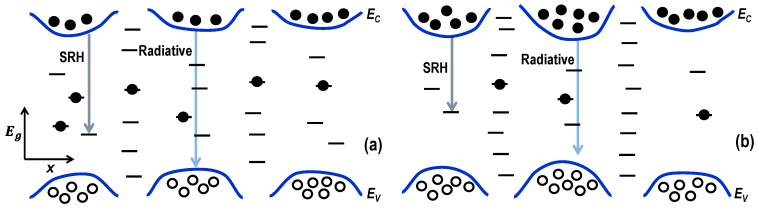
Schematic energy band diagram of the local in-plane potential fluctuation in the very small area of the QWs, where horizontal lines and filled (open) circles indicate the point defects and electrons (holes), respectively, with different levels of radiative and nonradiative recombination rates for samples (**a**) #1 and (**b**) #3.

Figure 5 depicts the proposed schematic energy band diagram of the local in-plane potential fluctuation in the QWs in a very small region of the active QWs. Point defects due to vacancies of atoms (Ga or N) are not uniformly distributed in the QW region. As neighboring atoms relax near the sites with accumulated defects, barriers are created around them and “valleys” with fewer defects come into existence. Figure 5a represents the case for sample #1 and Figure 5b for sample #3 as a higher strain and a stronger piezoelectric field in the active QW would induce more relaxation and, thus, deeper valleys and higher barriers. Since the potential barriers prevent the carriers from diffusing into the defects, carriers are localized at these potential-energy valleys [3,4,15,19,23]. The combined effect of the increase of the potential barrier caused by the piezoelectric field and the decrease of defects in the valley causes the variation in optoelectronic performances with the sample number. It should be noted that since the point defects change the distribution of In atoms over the QWs [51], the In concentration in potential-energy valleys could be higher than the accumulated-defect sites. The combined effects of the strain and the In concentration between defect sites would determine the depth of the potential-energy valley.

The difference in macroscopic optoelectronic characteristics of the same-wafer samples as shown in Table 1 can be consistently described by using the proposed local in-plane potential fluctuation model of the QWs. At low currents, carriers can have two different kinds of processes: (i) localization at the valleys that mostly causes the radiative recombination; and (ii) capture by the point defects (i.e., NRCs) that causes the SRH nonradiative recombination. As the current is increased from 5 × 10^−6^ A to ~1.5 × 10^−2^ A, the IQE of all samples increases due to the increment of the radiative recombination rate over the nonradiative recombination rate at the localized states. Beyond a certain current, the efficiency droop occurs due to a more rapid increase in the nonradiative recombination rate as carriers spill over to the defect sites and the p-GaN clad layer. The maximum IQE (as seen in Table 1) increases with the sample number, which is due to the reduced NRCs in the potential valleys of the QWs. At low currents, such as 5 × 10^−5^ A, the radiative recombination rate would increase with the sample number due to the influence of local in-plane potential fluctuation (deeper valley and, thus, higher carrier density) since more electron-hole pairs are available for radiative recombination.

As seen in Table 1, the IQE droop has a correlation with the piezoelectric field in the sample because the piezoelectric field in the MQWs decreases the overlap between the electron-hole wavefunctions, producing a reduced radiative recombination rate. The screening of the piezoelectric field by the injected carriers has been understood by the blue shift in the peak wavelength at low currents. However, at high currents, the red shifts in the emission spectra of samples #2 and #3 are increased with the increased IQE droop caused by the increased piezoelectric field, as mentioned above. Generally, this kind of red shift is due to the bandgap narrowing caused by the heat generation in the sample [35]: higher nonradiative currents with higher amounts of IQE droop increase the junction temperature of the samples, inducing higher red shifts in the emission spectra at high currents. It should be noted that the screening of the piezoelectric field by the injected carriers tends to decrease the wavelength (blue shift) by the QCSE, but this effect is not strong enough to prevent the red shift from occurring at high currents.

To keep the same device structure, size, growth condition, and substrate for all LED samples, we have selected all LEDs from the same wafer. Although the variations of optoelectronic performances of these LEDs are small, they have consistent interrelations between the macroscopic parameters. We have found that the improved crystal quality with the increased strain-induced piezoelectric field increases the IQE peak due to the increased local in-plane potential fluctuation in the QWs, which causes the localization of carriers in the less-defective energy valleys with the increasing radiative recombination rate.

## 4. Conclusions

In this paper, we have discussed the effects of local in-plane potential fluctuation in the InGaN/GaN QWs using three different blue LED samples from an identical wafer grown on a *c*-plane sapphire substrate through the analyses of interrelations between various macroscopic optoelectronic performances measured at room temperature. The increase in localized carriers in the less-defective energy valleys of the QWs leads to an increased peak IQE. The correlation between the slightly-increased strain in the samples and the carrier localization at the local energy valleys in the QWs emphasizes, once again, the importance of the strain-induced piezoelectric field in device performances for GaN-based LEDs grown on *c*-plane sapphire substrates. All the macroscopic characterizations examined in this paper indicate that the combined effects of the strain-induced piezoelectric field and point defects are behind the local in-plane potential fluctuation in the QWs for GaN-based LEDs. The present work sheds light on the significance of the local potential fluctuation in InGaN/GaN MQW LEDs from a different perspective and demonstrates how the device performance parameters are interrelated with the material parameters, complementing the conventional microscopic characterizations.

## Figures and Tables

**Table 1:** Summary of optoelectronic performances of the samples.

	$\eta_{\mathrm{IQE},\max}$ (%)	$I_{\max\;\mathrm{IQE}}$ (mA)	$\eta_{\mathrm{droop}}$ (%)	$n_{\mathrm{ideal}}$ (a.u.)	$I_{n}$ (mA)	$V_{\mathrm{FB}}$ (V)	$F_{\mathrm{PZ}}$ (MV/cm)	$\lambda_{p}$ (nm)
#1	89.0	15.0	15.0	1.52	0.20	−10.5	−1.35	435.5
#2	91.5	10.0	16.0	1.47	0.10	−10.9	−1.40	436.5
#3	92.0	9.0	16.6	1.45	0.09	−11.5	−1.50	438.0

*η*_IQE,max_ = peak (maximum) IQE, *I*_max IQE_ = current at the maximum IQE, *η*_droop_ = IQE droop, *n*_ideal_ = minimum ideality factor, *I*_n_ = current at the minimum ideality factor, *V*_FB_ = flat-band voltage (at which the strain-induced piezoelectric field is compensated), *F*_PZ_ = piezoelectric field, and *λ*_p_ = peak wavelength.
